# Virtual reality and augmented reality in medical education: an umbrella review

**DOI:** 10.3389/fdgth.2024.1365345

**Published:** 2024-03-14

**Authors:** Talia Tene, Diego Fabián Vique López, Paulina Elizabeth Valverde Aguirre, Luz María Orna Puente, Cristian Vacacela Gomez

**Affiliations:** ^1^ Department of Chemistry, Universidad Técnica Particular de Loja, Loja, Ecuador; ^2^ Escuela Superior Politécnica de Chimborazo (ESPOCH), Riobamba, Ecuador; ^3^ INFN-Laboratori Nazionali di Frascati, Frascati, Italy

**Keywords:** immersive technologies, virtual reality, augmented reality, medical education, medical training and learning

## Abstract

**Objective:**

This umbrella review aims to ascertain the extent to which immersive Virtual Reality (VR) and Augmented Reality (AR) technologies improve specific competencies in healthcare professionals within medical education and training, in contrast to traditional educational methods or no intervention.

**Methods:**

Adhering to PRISMA guidelines and the PICOS approach, a systematic literature search was conducted across major databases to identify studies examining the use of VR and AR in medical education. Eligible studies were screened and categorized based on the PICOS criteria. Descriptive statistics and chi-square tests were employed to analyze the data, supplemented by the Fisher test for small sample sizes or specific conditions.

**Analysis:**

The analysis involved cross-tabulating the stages of work (Development and Testing, Results, Evaluated) and variables of interest (Performance, Engagement, Performance and Engagement, Effectiveness, no evaluated) against the types of technologies used. Chi-square tests assessed the associations between these categorical variables.

**Results:**

A total of 28 studies were included, with the majority reporting increased or positive effects from the use of immersive technologies. VR was the most frequently studied technology, particularly in the “Performance” and “Results” stages. The chi-square analysis, with a Pearson value close to significance (*p* = 0.052), suggested a non-significant trend toward the association of VR with improved outcomes.

**Conclusions:**

The results indicate that VR is a prevalent tool in the research landscape of medical education technologies, with a positive trend toward enhancing educational outcomes. However, the statistical analysis did not reveal a significant association, suggesting the need for further research with larger sample sizes. This review underscores the potential of immersive technologies to enhance medical training yet calls for more rigorous studies to establish definitive evidence of their efficacy.

## 1 Introduction

The advent of virtual reality (VR) and augmented reality (AR) technologies has initiated a paradigm shift in the domain of medical education and training. This umbrella review meticulously deconstructs and synthesizes the extensive contributions of these technologies, examining their potential to significantly elevate learner performance/engagement and improve pedagogical outcomes. Informed by the PICOS framework, our research question aims to assess the extent to which immersive VR and AR technologies, compared to traditional educational methods or no intervention, enhance specific competencies in healthcare professionals within medical education and training contexts.

In this context, the integration of haptic feedback technology, as investigated by Kapoor et al. (1), marks an important advancement in medical training. The tactile engagement provided by haptic interfaces in virtual environments has transformed the development of surgical skills, offering a high-fidelity replication of clinical procedures. Concurrently, AR technologies, as elucidated by Herron et al. (2) and Rochlen et al. (3), have begun to redefine the educational environment, merging interactive experiences with practical applications in a manner that traditional didactic methods cannot.

The deployment of VR in medical training, highlighted by Nguyen et al. (4), Childs et al. (5), and Walbron et al. (6), demonstrated the value of this technology as an educational asset. VR offers a diverse range of applications—from enhancing ophthalmologic examinations to simulating arthroscopic procedures—suggesting a significant shift from conventional training methods to those that are more cost-effective, repeatable, and learner-friendly.

The role of immersive technologies in fostering empathy within medical training is underscored by Elzie et al. (7), who demonstrated the effectiveness of VR in bridging the cognitive and emotional gap between healthcare providers and patients. This empathetic approach is complemented by the work of Gloy et al. (8) and Lian et al. (9), who attested the ability of VR to improve the retention of complex anatomical knowledge, indicating a marked advantage over traditional learning techniques in terms of engagement and information recall.

The importance of visual communication in healthcare, emphasized by Yaqi et al. (10), grants the potential of VR to elucidate complex medical conditions and treatments, thereby enhancing patient and provider experiences. This facet is integral to the educational transformation VR and AR are spearheading, as these technologies facilitate a deeper understanding and communication of medical concepts. Also, the development of VR training systems specifically designed for anatomy education and medical procedures, as explored by Falah et al. (11) and Ruthenbeck et al. (12), underlined the ability of these systems to improve learning outcomes and procedural competencies. Lee et al. (13) and Alfalah et al. (14) further illustrated practical applications of AR in enhancing the visualization of anatomical structures, an essential feature for surgical planning and educational purposes.

The study by Ivanov et al. (15) and Alahmadi et al. (16) signaled a leap forward in medical education through the application of AR in emergency medicine simulations and the development of educational apps like CardioSim, respectively. These innovations present engaging and effective methodologies for learning complex medical subjects.

Considering the disruptions caused by the COVID-19 pandemic, Dhar et al. (17) assessed the role of AR in sustaining the continuity of medical education, while Khundam et al. (18) contributed to the ongoing dialogue by examining different VR interaction modalities, affirming that technology can effectively accommodate diverse learning preferences without compromising outcomes. Albrecht et al. (19) advocated for the incorporation of VR simulators into medical curricula, reinforcing the acquisition of intricate clinical skills such as otoscopy.

The efficacy of AR in surgical oncology training and procedures is evaluated by Prunoiu et al. (20), highlighting AR's potential to enhance surgical performance. Toohey et al. (21) delved into the psychological impacts of simulation training, suggesting AR can create emotionally realistic yet manageable training scenarios. Finally, Lin et al. (22) and McBain et al. (23) demonstrate the capability of AR to advance complex professional skills and refine anatomical dissection training. In general, all these works delineate a trajectory toward an educational future where immersive technologies are not merely adjunct tools but are deeply integrated into the fabric of medical training, shaping the competencies of healthcare professionals in profound and multifaceted ways.

Hence, considering rapidly evolving technological sceneries and emerging global health challenges, this umbrella review aims to consolidate diverse perspectives and findings, providing an in-depth and comprehensive analysis of the role and future potential of VR and AR technologies in medical education—a domain of the cusp of significant transformation. Hence, adhering to PRISMA guidelines, our systematic review conducts a thorough literature synthesis to elucidate the effectiveness of immersive virtual and augmented reality technologies in enhancing medical training. Framed within the PICOS approach, our research question has been fashioned as:

• “To what extent do immersive virtual reality and augmented reality technologies, compared to traditional educational methods or no intervention, improve specific competencies in healthcare professionals as part of medical education and training?”

This question guides our exploration into the efficacy of VR and AR technologies in medical education, focusing specifically on outcomes like the retention of medical knowledge, the development of problem-solving skills, and the enhancement of critical thinking abilities. Our systematic review aims to provide a decisive answer to these pivotal questions, thus offering valuable insights into the transformative impact of VR and AR on the competence of medical professionals in an ever-evolving healthcare landscape.

## 2 Methodology

In this study, we utilize the PICOS approach, a rigorously established and widely acknowledged framework for formulating precise and focused research questions within systematic reviews (24). The PICOS model methodically breaks down the research question into five essential components, each serving to distill and clarify the key aspects of the inquiry. These components are succinctly presented in Table 1, providing a structured overview of the research scope and direction, as follows:

• Population (P) has been specified to include both students and practicing healthcare professionals engaging in medical education and training.
• Intervention (I) mentions the use of VR and AR technologies in the educational process.
• Comparison (C) has been defined to include traditional educational methods or the lack of any intervention, which provides a baseline against which to measure the impact of VR and AR.
• Outcomes (O) have been detailed to reflect not just engagement or performance broadly, but specific competencies that are crucial in medical training.
• Study Design (S) has been expanded to include systematic reviews and meta-analyses, as these are the studies typically included in an umbrella review.

**Table 1:** The research question of the presented review is defined according to the PICO approach.

P	Population	Healthcare professionals in training (includes medical students and practicing professionals engaging in continuous education)
I	Intervention	Immersive virtual reality (VR) and augmented reality (AR) technologies used in medical education and training
C	Comparison	Traditional educational methods or no intervention
O	Outcome	Improvement in specific competencies (e.g., knowledge retention, procedural skills, diagnostic accuracy, decision-making)
S	Study Design	Systematic reviews and meta-analyses of studies evaluating the use of VR and AR in medical education

This framework serves as a valuable tool for making precise research questions and devising study designs that facilitate the efficient collection of pertinent evidence.

**Figure 1:**
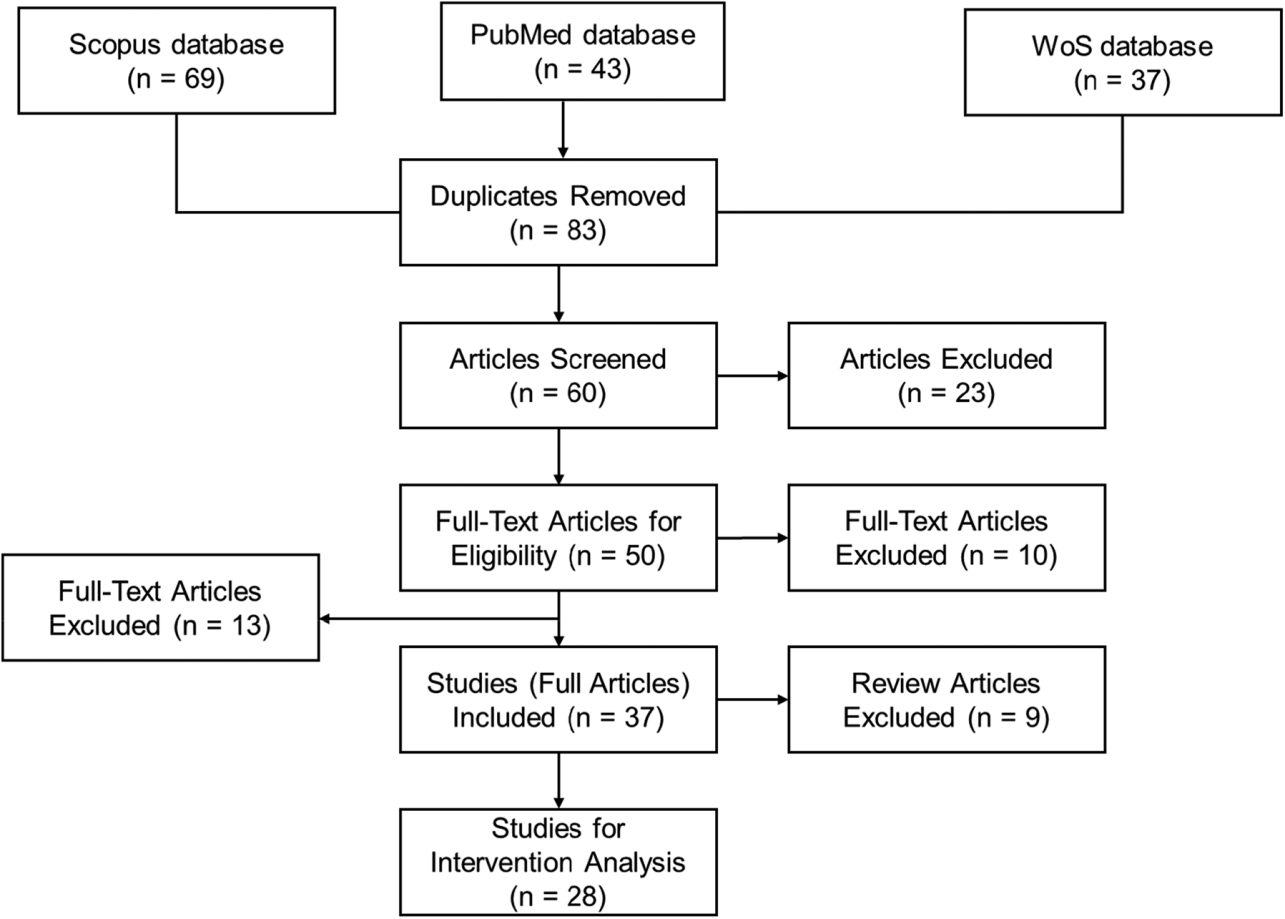
Flow chart of current systematic review.

Figure 1 delineates the methodological framework employed in our study, conforming to the PRISMA guidelines (25), and is characterized by a deliberate temporal boundary spanning from 2012 to 2022. This decade encapsulates a key period in the evolution and refinement of VR and AR technologies, marking significant milestones in their sophistication and integration into diverse domains. It is within this context that VR/AR technologies have seen substantial advancements in hardware and software capabilities, leading to enhanced realism and user experience. Moreover, this period is critical as it aligns with an increased adoption of these technologies in medical education and training, prompted by a growing recognition of their potential to enhance learning outcomes and simulate complex medical scenarios. Consequently, focusing on this transformative decade provides a robust understanding of the technological progression and educational implementation, offering a contemporary and relevant analysis of the impacts and advancements in the use of VR/AR in the medical field.

### 2.1 Identification

In May 2023, we executed a comprehensive search aimed at identifying pertinent literature for inclusion in this review. Our search strategy included three databases: Scopus, Web of Science, and PubMed. These platforms were selected for their extensive coverage, interdisciplinary content, and relevance to the medical and educational fields, thus ensuring a broad yet specific capture of literature about “*Exploration of Virtual Reality and Augmented Reality Applications in Medical Education and Training*”. Scopus offers a vast repository of peer-reviewed literature with a strong emphasis on scientific, technical, and medical content. Web of Science is known for its high-quality research articles and citation indexing, which facilitates the tracking of seminal work in the field. PubMed, a free resource developed by the National Center for Biotechnology Information (NCBI), is a premier database for biomedical literature from MEDLINE, life science journals, and online books.

These databases are preferred over other sources such as MEDLINE (partially indexed within PubMed and thus redundant), AMED (which focuses on allied health and complementary medicine, areas less central to our VR/AR focus), ERIC (which is education-focused but less comprehensive in medical training technologies), and CINAHL Plus (which concentrates on nursing and allied health, offering less scope in advanced medical education technologies). By harnessing the combined breadth and depth of Scopus, Web of Science, and PubMed, we ensure extensive coverage of the relevant literature without significant overlap, thereby streamlining our search process and maintaining a high standard of evidence selection.

These strategically targeted search results are summarized in Table 2, which presents a synthesis of the article number retrieved during this phase.

**Table 2:** Query type and the corresponding results.

Database	Query	Results
Scopus	(“VIRTUAL REALITY” OR “AUGMENTED REALITY”) AND (“MEDICAL EDUCATION” AND “MEDICAL TRAINING”)	69
Web of sciences	(“VIRTUAL REALITY” OR “AUGMENTED REALITY”) AND (“MEDICAL EDUCATION” AND “MEDICAL TRAINING”)	37
PubMed	(“VIRTUAL REALITY” OR “AUGMENTED REALITY”) AND (“MEDICAL EDUCATION” AND “MEDICAL TRAINING”)	43

Year restriction applied from 2012 to 2022.

### 2.2 Screening

After conducting a comprehensive database search, we identified a total of 149 relevant articles: 69 from the Scopus database, 45 from PubMed, and 37 from the Web of Science. At this stage, we excluded conference papers, non-English language publications, books, and book chapters to focus exclusively on peer-reviewed journal articles. After the removal of duplicates, 83 unique articles remained. These articles were then subjected to a rigorous screening process where titles and abstracts were reviewed according to the following inclusion criteria, in line with our research question and the PRISMA guidelines:

• Inclusion of only full or original research articles, thereby excluding editorials, letters, and comments.
• Consideration of articles specifically about medical education and training, to ensure direct relevance to the focus of the current study.
• Requirement for the articles to discuss the use of VR, AR, or Immersive Virtual Reality technologies, reflecting our interest in these specific educational tools.
• Inclusion of articles that explore the application of simulators or learning outcomes in a medical context, aligning with the intervention component of our PICOS framework.

These criteria led to the exclusion of 23 articles during the screening stage. The remaining 60 articles were deemed suitable for full-text review to assess eligibility for inclusion in the final review. Each article was evaluated in detail to determine whether it conformed to our methodological and content-specific standards, which were pre-defined to minimize bias and enhance the systematic nature of the review process.

### 2.3 Eligibility

During the Eligibility phase, a systematic and unbiased assessment of the full texts was conducted. Each article from the screening phase was assigned randomly to members of the research team to ensure a thorough examination. We adhered to the following specific eligibility criteria, informed by the key concepts of our research question and study design:

• The article must explicitly discuss the application of AR and VR technologies within the context of medical education.
• The focus should be on immersive virtual reality as a tool for medical learning, teaching, or practical application.
• The implementation of a virtual environment must be primarily for educational purposes within the medical field.

During this full-text review, 50 articles satisfied all the eligibility criteria and were selected for detailed data extraction and subsequent analysis. Hence, we excluded 10 articles that did not demonstrate the application of a virtual environment for educational purposes in medicine. Additionally, 13 further articles were excluded as they lacked observable interventions related to medical education and training, which is a critical component of our review scope. Consequently, a total of 23 articles were deemed ineligible for inclusion in the data synthesis phase. However, given their relevance to the broader context of VR and AR use in medicine, these articles were instrumental in shaping the introduction section of our systematic review. They provided valuable background information and helped to delineate the background of current research in this field.

Therefore, 37 articles satisfied the inclusion criteria and subsequent analysis. This stage was critical in ensuring that our review was grounded in the most relevant and rigorous evidence available.

### 2.4 Included

In the final inclusion phase, we meticulously performed data extraction from the articles that fulfilled all our eligibility criteria. This process was designed to capture detailed information on several key aspects of the studies:

• The impact of the intervention on student performance and/or engagement.
• The specific type of virtual technology utilized in the intervention.
• The effects observed because of the intervention.
• The number of participants and their roles within the study.

As stated, we identified 37 articles that were directly aligned with our research objectives and thus included them for comprehensive analysis. The data extraction criteria focused on:

• The design of the virtual reality or augmented reality tool and the planning of its content.
• The development of learning materials within the virtual environment.
• The cognitive load imposed by virtual technology and associated time management.
• The feedback mechanisms and level of interactivity offered by the technology.
• The distribution of participants in the studies, categorized by their roles and involvement.

It is important to note that within this cohort, we identified 9 articles that were review papers. Consistent with the PRISMA guidelines, these were excluded from the main analysis because they did not present original research data. Nonetheless, these review articles were invaluable for contextual comparison and discussion in Section 4.3 of our systematic review. Their inclusion in this comparative discussion section helped to elucidate the novel contributions of our work to the existing body of knowledge. Therefore, a focused subset of 28 articles was advanced to the subsequent stage of in-depth intervention analysis, as detailed in the following sections of our review.

In our commitment to transparency and reproducibility, we have made our raw data, data processing details, and a summary of each stage of our review process publicly available. This information can be accessed at doi: 10.17605/OSF.IO/P3TGJ. This repository includes all relevant data and methodological steps, offering an open and comprehensive view of our research process.

## 3 Results

### 3.1 Summary of search results

By the PRISMA guidelines and utilizing the PICOS approach, the results of the literature search and selection process for the systematic review on the impact of AR and VR in medical education and training are detailed in Figure 1. Indeed, a comprehensive initial search across three databases—Scopus, PubMed, and Web of Science (WoS)—yielded a total of 149 articles, with 69, 43, and 37 articles identified from each respective database. After the removal of duplicate articles (i.e., 83 articles were considered at the initial stage), the remaining 60 articles were screened based on their titles and abstracts. Screening resulted in the exclusion of 23 articles that did not meet the predefined criteria. The remaining 50 articles underwent full-text review for eligibility, of which 13 were excluded. Reasons for exclusion at this phase included lack of relevance to the application of virtual environments for educational purposes, absence of interventions in the context of medical education and training, or the article type being a review rather than original research.

Therefore, 37 full articles were included for data extraction. These articles explored a range of interventions involving AR or VR technologies in medical education and training, assessing their effects on the performance of healthcare professionals, engagement, or both. At this point, 9 review articles were excluded from the intervention analysis but were utilized for contextual comparison in the systematic review, given their importance in framing the current state of AR and VR in medical education.

The remaining 28 studies were specifically selected for intervention analysis, which entailed a deeper investigation into the types of VR/AR interventions and their direct impact on learners within the medical field. The data extraction process focused on key factors such as the design and content planning of the tools, the development of learning materials, cognitive load management, feedback provided, interactivity of the simulations, and participant numbers based on roles.

To further emphasize, the selected 28 studies were evaluated in the context of the impact of immersive VR and AR interventions within medical education. These studies were particularly chosen for their relevance to our research question, as follows:

• The impact of these interventions on performance is meticulously defined and measured as an enhancement in the skill acquisition and knowledge retention of medical practitioners. This encompasses a broad spectrum of outcomes, including improved proficiency in various medical procedures, heightened accuracy in diagnostic techniques, and an overall increase in the retention of complex medical information. This dimension of performance is critical as it directly relates to the competencies that healthcare professionals must acquire and refine throughout their careers.
• Engagement, as evaluated in these studies, encompasses the multifaceted ways in which medical students interact with and embrace these emerging technologies. It is characterized by an increased willingness among students to integrate VR and AR systems into their learning paradigm, actively participate in technology-driven educational activities, and exhibit a sustained interest in the ongoing advancement of these immersive tools. Engagement is a crucial metric as it is indicative of both the acceptance of these technologies in the medical community and their potential to sustain long-term changes in medical education methodologies.
• The impact of interventions on both performance and engagement is believed to be fundamental in determining the efficacy of immersive technologies in medical education. These factors, when enhanced, suggest a substantial improvement in the quality of training for future healthcare professionals and a progressive shift towards more interactive, personalized, and effective learning experiences.

This systematic review, therefore, analyzes the quantifiable effects of immersive technologies on medical education. Table 3 shows the distinct impact of VR and AR technologies. Specifically, of the 28 studies scrutinized, 16 exclusively investigate the performance enhancements attributed to these technologies, while 3 focus solely on assessing learner engagement. A further 5 studies ambitiously evaluate both engagement and performance. Additionally, 1 study delves into the whole effectiveness of these interventions, and 3 studies scrutinize the applications of immersive virtual reality tools without a direct evaluation of outcomes.

**Table 3:** Extracted structural elements with corresponding interventions and measured effect.

Stage	Intervention	Variable	Effect	IVT	No. participants	Ref.
Development and testing	Use the PhysX commodity physics engine, which aimed to create a two-handed interactive simulation system for medical training in suturing techniques	Performance	Positive	VR	3	Choi et al. (26)
Evaluation	Discussed is the application of haptic feedback to virtual reality video game training programs for emergency responders.	Effectiveness	Increased	VR	No specified	Gao et al. (27)
Development and testing	Development and implementation of a VR simulator designed to enhance ACLS training for healthcare providers.	Performance	Increased	VR	No specified	Vankipuram et al. (28)
Development and testing	Using a VR educational tool to visualize and interact with the anatomy of the human cranium for educational and surgical planning.	Engagement	Increased	VR	No specified	Izard et al. (29)
Evaluated	Using are the two systems developed for medical training: An interactive 360 content system that presents medical procedures in a virtual environment. An interactive virtual reality medical simulator that aids in understanding and performing medical procedures.	Performance	Increased	VR	16	Izard et al. (30)
Evaluated	Using are the two systems developed for medical training: An interactive 360 content system that presents medical procedures in a virtual environment. An interactive virtual reality medical simulator that aids in understanding and performing medical procedures.	Performance	Increased	VR	26	Yovanoff et al. (31)
Results	VR-based serious gaming module for Basic Life Support (BLS) training, combined with functional near-infrared spectroscopy (fNIRS) to monitor cerebral oxygenation levels.	Performance and Engagement	Increased	VR	22	Aksoy et al. (32)
Evaluated	Use of an immersive virtual reality consultation scenario to train doctors to recognize safeguarding cues related to child protection.	Performance	Positive	VR	63	Drewett et al. (33)
Results	Use of virtual reality (VR) simulation for teaching orthopedic surgery residents	Performance	Increased	VR	14	Hooper et al. (34)
Results	Development and use of a ray-tracing-based ultrasound simulation framework that incorporates real-time deformation and patient-specific scatterer maps to enhance the realism of ultrasound simulations.	Performance	Increased	VR	12	Starkov et al. (35)
Evaluated	Implementation of an online VR training platform (“Body Interact”) for medical students when in-hospital training was not possible due to the pandemic.	Engagement	Positive	VR	122	De Ponti et al. (36)
Evaluated	Use of VR-SBL in two case studies at the Universidad Europea de Madrid. One involved first aid training in a simulated traffic emergency, and the other involved simulating accidents in a virtual reality laboratory setting.	Performance and engagement	Increased	VR	No specified	Mariscal et al. (37)
Results	Use of an optical see-through augmented reality (OST-AR) training tool for central venous catheterization (CVC).	Performance	Positive	AR	18	Mendes et al. (38)
Results	The TACTICS VR training platform, which was developed as an evidence-based application to address the gap in stroke workflow optimization training.	Performance	Positive	VR	7	Hood et al. (39)
Evaluated	Using a Microsoft HoloLens-based AR system for surgical training and telementoring. This system uses 3D tracking and visualization to guide surgical procedures	Performance	Increased	AR	No specified	Liu et al. (40)
Evaluated	Training participants using different methods: VR-based instructions, video-based instructions, and paper-based instructions for setting up a surgical robot.	Performance and engagement	Positive	VR	30	Mehrfard et al. (41)
Evaluated	Use of an AI-based virtual reality (VR) trainer designed to simulate operating room (OR) fire scenarios as part of the training curriculum.	Performance	Increased	VR	53	Qi et al. (42)
Results	Use of AR step-by-step guide developed for the Microsoft HoloLens 2 for ECMO cannulation training, as opposed to conventional training methods.	Performance and Engagement	Increased	VR	21	Wolf et al. (43)
Evaluated	Use of virtual reality (VR) in medical education, particularly during the COVID-19 pandemic where traditional education methods were disrupted.	No evaluated	Positive	VR	No specified	Syed Abdul et al. (44)
Results	The application of extended reality within the metaverse for health communication, aiming to enhance health behavior change and address challenges in health communication.	No evaluated	Positive	VR	No specified	Piechata et al. (45)
Evaluated	Use of a desktop virtual reality application designed to teach undergraduate nursing students the ISBAR approach for preoperative assessment.	Performance and Engagement	Increased	VR	9	Andreasen et al. (46)
Evaluated	AEducaAR tool, which is an AR-based learning tool combining AR technology with 3D printing to teach human anatomy.	Performance	Neutral	AR	62	Cercenelli et al. (47)
Results	Creation and use of a scenario-based, mixed-reality (MR) platform for training non-technical skills (NTS) of battlefield first aid (BFA).	Performance	Increased	AR/VR	20	Du et al. (48)
Development and testing	The proposal and development of the metaverse for healthcare, termed MeTAI, which encompasses applications such as virtual comparative scanning, raw data sharing, augmented regulatory science, and metaversed medical intervention	No evaluated	No evaluated	AR/VR	No specified	Wang et al. (49)
Results	Use of an augmented reality (AR)-based self-learning system for setting up mechanical ventilators as compared to a manual-based instruction system.	Performance	Increased	AR	31	Heo et al. (50)
Evaluated	Use of a semi-autonomous virtual reality (VR) trauma simulator for medical education	Engagement	Increased	AR/VR	17	Lombardo et al. (51)
Results	Application of MR technology to create 3D holographic models for use in orthopedic surgery planning and navigation.	Performance	Positive	AR/VR	No specified	Lu et al. (52)
Development and testing	Use of a VR application for training medical students in COVID-19 swab testing and proper handling of personal protective equipment (PPE)	Performance	Positive	VR	29	Zikas et al. (53)

The collective insights gleaned from this body of work underscore a predominant trend: an observable enhancement in educational outcomes linked to the integration of VR and AR in medical training. These interventions are consistently correlated with positive trends in both the acquisition of medical competencies and the active participation of students in their educational journeys. Nevertheless, there is an outlier; one study presents a neutral effect, which may stem from various factors that require further exploration to fully understand the implications of this finding on the broader application of immersive technologies in medical education. The nuances of this singular result serve as a reminder of the complexity inherent in educational interventions and the need for a multifaceted approach to their assessment and interpretation.

### 3.2 Summary of interventions

The synthesized data from Table 3 of the systematic review illustrates the outcomes of interventions involving VR and AR technologies in medical education, as delineated by distinct evaluative criteria:

• Regarding “Performance”, 16 studies were examined, revealing a predominantly positive impact. Of these, six reported a positive effect, one a neutral effect, and the remaining nine noted an increased effect on performance metrics. This suggests that the integration of VR and AR technologies in medical training has a generally favorable outcome on the performance capabilities of healthcare professionals.
• In the area of “Engagement”, three studies were evaluated. One of these studies observed a positive effect, while the other two reported an increased level of engagement among medical students and professionals. This enhancement in engagement indicates a promising shift towards more immersive and interactive learning environments facilitated by these technologies.
• For the combined metrics of “Performance and Engagement”, five studies were scrutinized, with one study indicating a positive effect and four showing an increased effect. This underlines the dual benefit of VR and AR technologies in simultaneously enhancing the learning process and the retention of knowledge and skills among medical trainees.
• One study focused on “Effectiveness”, which also reported an increased effect, reinforcing the notion that VR and AR interventions are not only engaging and performance-enhancing but also effective in their intended outcomes.
• Three studies were categorized as “No evaluated” due to their speculative or forward-looking nature. These studies are primarily perspective articles that, while not empirically evaluating outcomes, suggest potential positive effects of virtual technologies in two papers, with one work not assessing the effect.

These perspectives contribute to the broader understanding of the potential and future directions of immersive technologies in medical education, as follows:

• Choi et al. (26) developed a virtual suturing simulator that positively impacted medical training in suturing skills. This innovative tool integrated physics, graphics, and haptics simulation, improving user performance and offering a more efficient training method.
• Gao et al. (27) provided a case study that showed the effectiveness of haptic feedback in emergency responder training programs. This study demonstrated enhanced skill transfer to real-world situations for combat medics and civilian first responders, marking an advancement in medical simulation training.
• Vankipuram et al. (28) created an immersive VR simulator for Advanced Cardiac Life Support training. The simulator, which provided CPR feedback and recorded task-specific information, allowed for multi-user participation and remote learning, increasing the performance of healthcare providers in ACLS training.
• Hooper et al. (34) presented evidence of the effectiveness of VR simulation in improving orthopedic surgical skills of residents. This study showed that VR simulation could be a valuable addition to surgical education curricula, enhancing the training process for complex surgical procedures.
• Aksoy et al. (32) demonstrated the use of functional Near Infrared Spectroscopy (fNIRS) as a valuable tool for monitoring cognitive workload and assessing performance during VR-based Basic Life Support training. This integration improved learning outcomes and reduced cognitive workload, thus enhancing both performance and engagement in medical training.

Regarding the definition and application of VR in medical education, we admit the extreme inclusion of the study by Andreasen et al. (46). However, it is important to note that our systematic review adopts a comprehensive and inclusive approach to VR, recognizing that VR technology encompasses a spectrum of applications, from fully immersive environments to desktop simulations. The study by Andreasen et al., which evaluates a desktop-based VR application for preoperative ISBAR training, falls within this scope. This inclusion is supported by the fact that VR in education is an evolving field, with varied implementations that all serve the common goal of enhancing learning outcomes.

### 3.3 Data analysis

**Figure 2:**
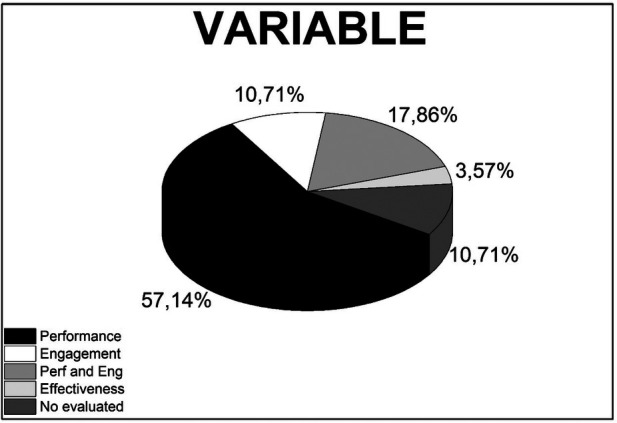
Distribution of studies analyzing the variables on performance, engagement, and both performance and engagement (perf and Eng), effectiveness, and no evaluated in medical education utilizing VR and AR technologies.

Figure 2 elucidates the distribution of studies examining the influence of VR and AR technologies on medical education across different variables. Performance, as a standalone metric, was the predominant focus of the studies, comprising 57.14% of the research. This prevalence underscores the criticality of evaluating the concrete skills and knowledge that learners acquire when engaged with immersive technologies. Engagement, reflecting the degree to which learners find the VR and AR experiences interactive and compelling, accounted for 17.86% of the studies, signifying a substantial interest in the user experience aspects of educational technology.

A combined analysis of both performance and engagement was undertaken in 10.71% of the studies, reflecting an integrated approach to gauge the comprehensive effect of these technologies on learners—both in terms of skill acquisition and interactive participation. Effectiveness, potentially encompassing broader aspects such as the efficiency and practical utility of VR and AR, was also scrutinized in 10.71% of the studies, pointing to a concern for the general value and applicability of these technologies in medical education. The remaining 3.57% of studies did not assess these outcomes, which may suggest potential gaps in the current literature or deliberate exclusions where the impact of VR and AR was deemed not applicable or beyond the objectives of the respective studies. This fraction could represent an opportunity for future research to explore uncharted dimensions of VR and AR applications in medical training and education.

**Figure 3:**
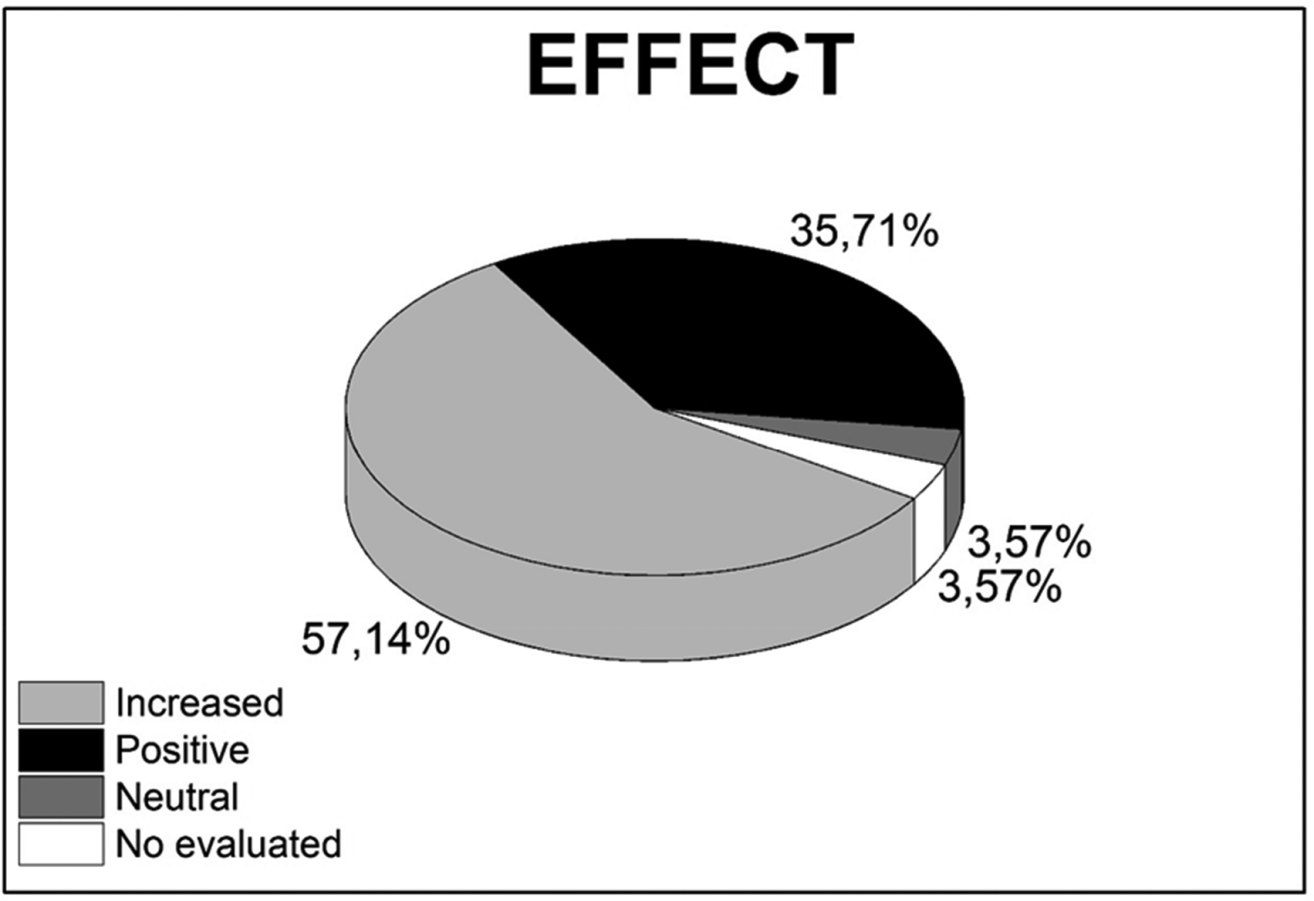
Distribution of studies analyzing the effect on increase, positive, neutral, and No evaluated in medical education utilizing VR and AR technologies.

Figure 3 provides a visual breakdown of the observed effects from studies assessing the impact of VR and AR technologies in medical education. The pie chart reveals that a majority, 57.14% of studies, reported an ‘Increased’ effect, which signifies a substantial enhancement in the measured outcomes due to the application of these technologies. A “Positive” effect is reported in 35.71% of the studies, suggesting beneficial outcomes that, while perhaps not quantified, still indicate favorable changes attributable to the use of VR and AR. The “Neutral” category, accounting for 3.57% of the studies, implies that in these instances, VR and AR did not significantly alter the measured variables. Equally, at 3.57%, the “No evaluated” portion suggests that some studies did not measure or report specific outcomes, which could point to unexplored areas for future research or aspects that were outside the primary focus of the study.

This distribution highlights the overwhelmingly positive reception of VR and AR technologies within medical education, with most studies recognizing their capacity to improve educational outcomes. The small percentage of studies with neutral or no evaluated effects indicates that while the general trend is towards benefit, there is room for a more nuanced exploration of the conditions under which these technologies may be most effective.

### 3.4 Descriptive analysis

The use of chi-square tests in the context of a systematic review is a methodological innovation that enhances the traditional narrative synthesis with a quantitative assessment of categorical data. While systematic reviews commonly aggregate findings qualitatively, the application of chi-square tests allows for a statistical exploration of the relationships between categorical variables such as the stage of work, variables studied, and effects observed in studies utilizing immersive virtual technologies.

In our systematic review, we elected to employ descriptive statistics to quantify the frequencies of various categories, as presented in Table 4. The chi-square test is particularly suitable for this analysis as it determines whether there is a significant association between two categorical variables, which, in our case, are the stages, variables, and effects related to VR and AR technologies in medical education. When the expected frequencies are too low to meet the chi-square test assumptions, The Fisher test offers a more accurate assessment, thereby ensuring the robustness of our statistical analysis even with smaller sample sizes. Hence, the rationale for selecting a *p*-value of less than 0.05 is grounded in conventional statistical practice, which considers this level as the cutoff for rejecting the null hypothesis—that there is no association between the variables. By setting this threshold, we assert with 95% confidence that the observed relationships in our study are not merely the result of random variation but may reflect genuine correlations.

**Table 4:** Prevalence of stage, variable, and effect in studies analyzed.

	AR (*n* = 4) %	VR (*n* = 20) %	AR/VR (*n* = 4) %	TOTAL (*n* = 28) %
Stage				
Development and testing	0.0	20.0	25.0	17.9
Results	50.0	30.0	50.0	35.7
Evaluated	50.0	50.0	25.0	46.4
Variable				
Performance	100.0	50.0	50.0	57.1
Engagement	0.0	10.0	25.0	10.7
Performance and engagement	0.0	25.0	0.0	17.9
Effectiveness	0.0	5.0	25.0	7.1
No evaluated	0.0	10.0	0.0	7.1
Effect				
Positive	25.0	40.0	25.0	35.7
Increased	50.0	60.0	50.0	57.1
Neutral	25.0	0.0	0.0	3.6
No evaluated	0.0	0.0	25.0	3.6

In the context of immersive virtual technologies versus Stage, we observe that:

• “Development and Testing” stage had 5 instances, with VR being the most used technology (4 times), followed by AR/VR (1 time), and AR was not used.
• In the “Results” stage, there were 10 instances. VR was again the most prevalent (6 times), followed by both AR and AR/VR being used twice.
• For the “Evaluated” stage, there were 13 instances where VR was used 10 times, indicating it is the most used technology in this stage, with AR and AR/VR being used less frequently (2 times for AR and once for AR/VR).
• General, VR was the most frequently reported technology (20 times), followed by AR (4 times) and AR/VR (4 times) across all stages.
• The Pearson Chi-square value is 1.982 with 4 degrees of freedom and a *p*-value of 0.739.
• The Likelihood ratio is 2.720 with 4 degrees of freedom and a *p*-value of 0.606.
• The Linear-by-Linear Association has a value of 0.859 with 1 degree of freedom and a *p*-value of 0.354.

Based on the chi-square test results, we conclude that there is no statistically significant relationship between the stages of work in medical education and the type of immersive technology used. VR appears to be the most utilized technology across all stages, but this usage does not statistically differ from the use of AR or combined AR/VR technologies in the context of the stages of work considered in this systematic review.

For immersive virtual technologies versus Variables, we point out that:

• The cross-tabulation displays the distribution of studies by the variable of interest (Performance, Engagement, Performance and Engagement, Effectiveness, No evaluated) against the type of immersive technology used (AR, VR, AR/VR).
• “Performance” is the most studied variable (16 instances in total), with VR being the most used technology (10 out of 16 times), followed by AR (4 times), and AR/VR (2 times).
• “Engagement” was studied less frequently (3 instances), with VR being used in 2 studies and AR/VR in 1 study. AR was not used for studies focusing solely on engagement.
• “Performance and Engagement” were jointly studied in 5 instances, with VR being used in all 5 studies.
• “Effectiveness” was evaluated in 2 instances, with both VR and AR/VR represented once.
• There were also 2 instances where the variable was “No evaluated,” with AR and VR each being used once.
• The Pearson Chi-square statistic is 7.700 with 8 degrees of freedom. The corresponding *p*-value is 0.463.
• The Likelihood Ratio statistic is 9.194 with 8 degrees of freedom. The *p*-value for this test is 0.326.
• The Linear-by-Linear Association statistic is 1.150 with 1 degree of freedom and a *p*-value of 0.284.

The distribution of studies according to the variable of interest and the type of immersive technology suggests a tendency towards VR in assessing both performance and engagement. However, the chi-square test indicates that these tendencies are not statistically significant. In essence, there appears to be no preferential association between the variables studied and the type of immersive technology used according to the sample of studies included in this review.

Finally, for immersive virtual technologies versus Effect, we show that:

• The cross-tabulation provides a breakdown of the effects (Positive, Increased, Neutral, No evaluated) observed in studies utilizing AR, VR, and AR/VR technologies.
• A “Positive” effect was reported in 10 instances, with VR having the highest frequency (8 times), followed by AR and AR/VR (1 time each).
• An “Increased” effect was the most common, with 16 instances. VR again had the highest frequency (12 times), AR had 2, and AR/VR also had 2.
• A “Neutral” effect was least common, with only one instance, which was associated with AR.
• There was 1 instance of “No evaluated” effect, which was associated with AR/VR.
• The Pearson Chi-square value is 12.460 with 6 degrees of freedom and a *p*-value of 0.052.
• The Likelihood Ratio value is 8.273 with 6 degrees of freedom and a *p*-value of 0.219.
• The Linear-by-Linear Association value is 0.255 with 1 degree of freedom and a *p*-value of 0.614.

The chi-square test results, particularly the Pearson Chi-square, suggest a potential pattern or trend in the data regarding the association between immersive technology type and observed effects, although this association does not reach conventional levels of statistical significance. There appears to be a stronger presence of “Increased” effects in studies involving VR, and “Positive” effects are also most frequently reported with VR. However, based on the current sample of studies, we cannot definitively conclude that there is a statistically significant association between the type of technology and the effect observed. Further research with larger sample sizes might be able to provide more definitive conclusions.

## 4 Discussions

### 4.1 Intervention contributions

It is important to focus on the diversity and impact of the interventions within the field of VR and AR in medical education, which are highlighted as follows:

• Choi et al. (26): This study represents a significant leap in medical training technology, particularly in suturing skills. The use of the PhysX physics engine to create a highly interactive simulation underscores the growing importance of realism in medical simulations. This work demonstrates the potential for VR to enhance fine motor skill development in medical professionals.
• Gao et al. (27): The application of haptic feedback in combat medic training is a pioneering contribution, particularly in enhancing the transfer of virtual training to real-world situations. This approach significantly improves the authenticity and effectiveness of training programs, potentially leading to better-prepared medics and first responders.
• Vankipuram et al. (28): The development of a VR ACLS simulator is notable for its emphasis on multi-user participation and remote learning capabilities. This study is a testament to the evolving nature of VR applications in medical training, where collaboration and distance learning are increasingly valued.
• Izard et al. (30): This research extends the utility of VR in medical education by allowing for repeated practice of surgical procedures. The development of interactive systems for understanding and performing medical procedures is a crucial step in automating surgical training and enhancing skill retention.
• Aksoy et al. (32): The integration of fNIRS technology for monitoring cognitive workload in VR-based training is a novel approach. This study contributes significantly to understanding how VR can be used to optimize learning by managing cognitive load, an aspect crucial for effective medical education.
• Mendes et al. (38): The validation of the OST-AR tool for CVC training represents an advancement in AR application in medical training. Its high acceptability and potential to enhance training without direct instructor intervention point to the future of autonomous learning tools in medical education.
• Wolf et al. (43): This exploration of AR in ECMO cannulation training illustrates the potential of this technology to standardize training and reduce errors in complex medical procedures. The emphasis on improving the clarity of information and stimulation during training marks a significant step forward in medical education technology.

These studies reflect the dynamic and evolving nature of VR and AR in medical education. They demonstrate how these technologies can enhance both the technical and cognitive aspects of medical training, from basic procedural skills to complex decision-making and problem-solving abilities. The integration of immersive technologies in medical education is not just about replicating real-world scenarios but also about enhancing and streamlining the learning process through innovative and interactive approaches.

### 4.2 Advantages

We have identified potential advantages of using immersive virtual technologies in medical education and training:

• Enhanced Realism and Immersion: Virtual technologies provide a highly immersive and interactive environment. For instance, Choi et al. (26) developed a virtual suturing simulator, and Izard et al. (29), a VR tool for studying human anatomy. Both works demonstrated how VR can create realistic settings for medical training, enhancing the understanding of complex procedures and anatomical structures.
• Improved Skill Transfer: The inclusion of haptic feedback in VR simulations, as explored by Gao et al. (27), aids in the effective transfer of skills learned in a virtual environment to real-world scenarios, which is critical in fields like combat medic training.
• Remote and Collaborative Learning: Virtual reality technology allows for remote learning and collaboration among medical trainees, breaking down geographical barriers and enabling a more inclusive and accessible education (28).
• Repeatable and Safe Practice: VR offers the opportunity for repeated practice in a risk-free environment. This is especially beneficial for procedures that cannot be frequently practiced on real patients (30, 31).
• Cognitive Load Management: Tools like fNIRS integrated into VR training, as discussed by Aksoy et al. (32), enable monitoring and managing the cognitive workload of learners, ensuring that the training is both effective and not overwhelming.
• Innovative Training Methods: Augmented reality and AI integration in medical training, as seen in the works of Mendes et al. (38) and Qi et al. (42), offer new ways to train medical professionals, enhancing their learning experience with innovative approaches.
• Error Reduction and Standardization in Training: AR can reduce errors in complex medical scenarios and standardize training performance, as demonstrated by Wolf et al. (43) in ECMO cannulation training.
• Adaptability and Flexibility in Learning Environments: The flexibility of VR and AR technologies to adapt to various learning needs and scenarios, as highlighted in the works of Syed Abdul et al. (44) and Lombardo et al. (51), makes them invaluable tools in medical education.
• Enhanced Engagement and Motivation: The interactive nature of VR and AR, shown in studies like those of Mariscal et al. (37) and Cercenelli et al. (47), fosters greater student engagement and motivation, leading to potentially better learning outcomes.

These advantages demonstrate the substantial impact that virtual technologies can have in revolutionizing medical education, making learning more interactive, effective, and adaptable to various needs and scenarios.

### 4.3 Comparison with previous reviews

Our systematic review, as delineated in Table 5 (This Review), advances the discourse on immersive technologies within the realm of medical education by offering a complete examination that includes both AR and VR. Diverging from prior reviews (54–62) that may have concentrated on singular technology or a broader educational purview, our review distinctively provides a side-by-side evaluation of the influence of AR and VR on performance (P) and engagement (E)—critical elements for efficacious learning. These analyses are particularly salient, delving into the adaptability of these technologies to fulfill the unique pedagogical requirements and learning targets specific to medical training.

**Table 5:** Comparison with previous review papers.

Refs.	AR	VR	Education (Y/N)	Field	Variable	Effect
Barsom et al. (54)	X	–	Y	Training healthcare.	P	Increased
Tatar et al. (55)	–	X	Y	Urology and general surgery	P	Positive
Myint et al. (56)	–	X	Y	Health professions education	P, E	Increased
Herur et al. (57)	X	X	Y	XR technologies.	P	Positive
Viglialoro et al. (58)	X	X	Y	Medical Education.	P	Positive
Pallavicini et al. (59)	–	X	Y	Treatments	P, E	Positive
Vayssiere et al. (60)	–	X	Y	Neurosurgery	P	Positive
Clarke et al. (61)	–	X	Y	Orthopedic surgery	P	Increased
Bansal et al. (62)	X	X	Y	Training healthcare	P	No evaluated
This review	X	X	Y	General	P, E, NE, EF	Positive/Increase/Neutral/No evaluate

P, performance; E, engagement; NE, no evaluated; EF, effectiveness.

Our systematic review rises above a general assessment of technological efficacy, instead focusing on attributes such as performance optimization and engagement deepening, along with their resultant impacts. Such a focus is indispensable in medical education, where the cultivation of practical skills and sustained engagement are essential for the realization of favorable educational outcomes.

### 4.4 Restrictions

This systematic review admits certain constraints inherent to the scope and methodology of academic research. The burgeoning quantity of literature on VR and AR within medical education presented a challenge, raising the possibility of inadvertently omitting relevant studies. The meticulous crafting of search queries and selection of keywords was decisive to the comprehensiveness. While the “snowballing” method was instrumental in uncovering pertinent keywords and studies, significant contributions may have been missed due to the imposed limitations and time constraints inherent in the research process.

Additionally, the review was bound by selective inclusion criteria, particularly the focus on peer-reviewed journal articles published in English. This restriction may have led to the exclusion of seminal research conducted in other languages or significant findings disseminated through non-journal outlets, potentially skewing the representation of the field's international breadth and diversity. To surmount these limitations and cultivate a richer synthesis of knowledge, subsequent inquiries should contemplate an expansion of the inclusion parameters. Incorporating studies in multiple languages and integrating grey literature, such as conference proceedings, theses, and technical reports, could yield a more holistic panorama of the worldwide progress and deployment of AR/VR technologies in medical education. Such a widened scope would not only enhance the universality of the systematic review's findings but also provide a more inclusive map of the field's innovation and practice.

### 4.5 Recommendations for future research

The adoption of VR and AR in medical education calls for a globally recognized framework that delineates essential elements of VR and AR applications within a learner-centric pedagogical model. This framework should articulate core standards and a cohesive understanding of their implementation in medical pedagogy. This framework must maintain flexibility to accommodate ongoing technological progress and emerging insights from educational psychology.

Current research paves the way for an in-depth exploration into the seamless incorporation of these innovative teaching methodologies into the fabric of VR/AR-enhanced medical education. Future studies should endeavor to elucidate how VR and AR technologies can not only heighten student engagement but also facilitate customized learning journeys, tailoring educational experiences to cater to the diverse needs and learning preferences of individual students.

As VR and AR technologies become more entrenched in the educational landscape, it is crucial to navigate the accompanying ethical considerations and privacy concerns. Subsequent research should rigorously examine these aspects, with a particular focus on the ethical handling of simulated patient data and the implications of using such technologies in student evaluation and training. It is essential to ensure that VR and AR integration into medical education adheres to ethical norms and maintains the sanctity of confidentiality.

Moreover, a meticulous evaluation of the array of VR and AR applications currently employed in medical education is vital. Such assessments should aim to discern the efficacy of various platforms and systems relative to specific educational objectives and user needs. Establishing a repository of best practices and identifying the most impactful educational technologies will serve as a critical resource for educators and institutions. This guidance is fundamental for informed decision-making regarding the incorporation of VR and AR into medical curricula, ensuring that these tools are harnessed to their full potential for the enhancement of medical education.

## 5 Conclusions

This umbrella review systematically examined the use of VR and AR in medical education to determine their effectiveness in enhancing the competencies of healthcare professionals compared to traditional methods. Through a methodical application of PRISMA guidelines and the PICOS approach, 28 studies were evaluated using descriptive statistics and chi-square analysis to understand the impact of immersive technologies on educational outcomes. Our findings suggest that VR is the most researched.

Technology, with a significant portion of the studies reporting enhanced performance outcomes when VR is employed. The studies included in this review have shown a trend where VR and AR technologies are positively associated with improved learner engagement and performance.

However, the statistical analysis did not provide evidence of a significant association between the stages of medical education and the type of technology used. This indicates that while there is a positive trend towards the effectiveness of VR and AR in medical education, it is not yet statistically significant within the body of literature reviewed. It is important to note that the lack of significance may be attributable to the diverse methodologies, small sample sizes, and the heterogeneity of outcomes across the included studies.

The neutrality and non-evaluation of effects in some studies point to potential areas for further research. These gaps highlight the necessity for more rigorous, standardized, and larger-scale studies to conclusively determine the impact of VR and AR technologies in medical education. While immersive VR and AR technologies demonstrate promise in enhancing medical training, more comprehensive research is required to establish their definitive effectiveness. Future investigations should aim to overcome the limitations observed in this review, focusing on consistent reporting, larger sample sizes, and possibly meta-analytic approaches to provide a more robust assessment of these innovative educational tools.

Finally, a critical aspect to consider is the complexity and cost associated with VR technology. Despite a downward trend in prices, equipping each student with individual VR/AR systems for remote learning remains a complex and expensive endeavor. This reality points towards a potential model where future remote learning could occur in semi-centralized labs equipped with VR/AR technologies. In these settings, students could physically access the necessary equipment, while educators could engage and instruct remotely. This approach could offer a more feasible and cost-effective solution for integrating immersive technologies into medical education, especially in settings where individual ownership of such devices is impractical.

## Data Availability

The raw data supporting the conclusions of this article will be made available by the authors, without undue reservation.
